# Selective Constraint on Copy Number Variation in Human Piwi-Interacting RNA Loci

**DOI:** 10.1371/journal.pone.0046611

**Published:** 2012-10-04

**Authors:** David W. Gould, Sergio Lukic, Kevin C. Chen

**Affiliations:** BioMaPS Institute for Quantitative Biology and Department of Genetics, Rutgers, The State University of New Jersey, Piscataway, New Jersey, United States of America; University of Miami, United States of America

## Abstract

Piwi-interacting RNAs (piRNAs) are a recently discovered class of small non-coding RNA found in animals. PiRNAs are primarily expressed in the germline where their best understood function is to repress transposable elements. Unlike previous studies that investigated the evolution of piRNA-generating loci at the level of nucleotide substitutions, here we studied the evolution of piRNA-generating loci at the level of copy number variation (i.e. duplications and deletions) using genome-wide copy number variation data from three human populations. Our analysis shows that at the level of copy number variation there is strong selective constraint and a very high mutation rate in human piRNA-generating loci. Our results differ from a model of positive selection on copy number variation in piRNA-generating loci previously proposed in rodents. We discuss possible reasons for this difference based on the transposable element insertion histories in the rodent and primate lineages.

## Introduction

Piwi-interacting RNAs (piRNAs) are an abundant class of 26–30-nt non-coding RNA, related to, but distinct from, microRNAs (reviewed in [1]). The best understood function of piRNAs is to repress transposable elements in the germline through a positive feedback loop called the “ping pong cycle” in which both sense and anti-sense transposable element transcripts are cleaved [2]. PiRNAs and transposable elements are expected to co-evolve in a Red Queen scenario and consequently have fast rates of evolution (reviewed in [3]). It is known that the piRNA pathway is ancient because Piwi proteins, which are a defining component of the piRNA pathway, and piRNAs are found in basal metazoans [4].

There have been a number of previous studies on piRNA evolution. A study of piRNA evolution focusing on single nucleotide polymorphisms in humans showed that piRNA-generating loci are evolving under selective constraint, particularly in African populations [5]. This result is consistent with the previous observation that transposable element insertion rates are higher in African compared to non-African populations [6] together with a model in which natural selection on piRNA-generating loci should be stronger in a population with a higher transposable element insertion rate. Computer simulations of piRNA-transposable element co-evolution in *Drosophila* suggested that piRNAs might allow transposable elements to reach high frequency in the population because the piRNAs attenuate the deleterious effects of transposable element insertions [7]. It is known that the sequences of piRNAs are poorly conserved between closely related species, such as mouse and rat [8], human and chimpanzee [5] or *C. elegans* and *C. briggsae* [9]. Note that many molecular features of *C. elegans* piRNAs (also called 21U RNAs), such as their biogenesis, differ from mammals and *Drosophila* [9]. The genomic locations of some piRNA-generating loci are conserved between species [10] but there are also many new piRNA-generating loci in rodents [8]. A number of piRNA pathway genes are also under positive selection [11], some times at the level of codon bias [12], consistent with a role for these genes in repressing transposable elements together with a model of piRNA-transposable element co-evolution.

PiRNA-generating loci are often found in large genomic clusters (called “piRNA clusters”) from which long primary transcripts are transcribed and individual piRNAs processed. In a previous study of piRNA cluster evolution between mouse and rat, Assis and Kondrashov [8] suggested that there is positive selection for an increased number of piRNA clusters in rodents since there are many duplications of piRNA clusters but few deletions. They observed that the rate of copy number evolution of piRNA clusters is much faster than for other gene families. However, their analysis was limited by the lack of usable copy number variation data in mouse or rat, which prevented them from carrying out a version of the McDonald-Kreitman test to conclusively prove the hypothesis of positive selection [8]. In particular, their analysis left open the possibility that mutational biases as opposed to natural selection could be driving the increase in piRNA cluster number in rodents. Here we addressed this question by studying the evolutionary forces acting on human piRNA-generating loci at the level of copy number variation using a comprehensive set of human CNV data.

## Results

We obtained human piRNA sequences from Girard *et al.* [10] and mapped them to the human genome as previously described (Methods). We also obtained human copy number variation (CNV) data from a recent paper by Conrad *et al.* [13]. The Conrad *et al.* study ascertained CNVs in Europeans (CEU) and Yorubans (YRI) and then genotyped the CNVs in these two populations as well as a population of Chinese and Japanese (CHBJPT). Our preliminary analysis of the Chinese and Japanese derived allele frequency spectrum (Figure S1) showed patterns that were difficult to interpret based on population genetics theory. In particular, there were many CNV segregating at intermediate allele frequency and in some classes of sites there were more CNVs in the highest frequency bin than in the lowest frequency bin. We attributed these unusual patterns in the site frequency spectrum to ascertainment biases stemming from the ethnic composition of the ascertainment panel. Thus we confined the remainder of our analysis to only the European and Yoruban data.

### PiRNA-generating Locihave a High Mutation Rate for Copy Number Variation

We first studied the rate of CNV formation in different regions of the human genome. A much higher proportion of piRNA-generating loci were in CNVs (12.0%) compared to intergenic regions (1.4%) that do not contain piRNA-generating loci. For the rest of the manuscript when we say “intergenic region” we mean intergenic region that do not contain any piRNA-generating loci, though the latter constitute only a very small fraction of all intergenic regions. More formally, any CNV that overlaps a gene is considered a genic CNV, any remaining CNV that overlaps a piRNA is considered a piRNA CNV and any remaining CNVs are considered intergenic CNVs. The excess of CNVs in piRNA-generating loci was much higher than even repeat regions of the genome annotated by RepeatMasker [14] (5.4%). For these results, we used the complete list of CNVs discovered in Conrad *et al.* [13], not the smaller list of CNVs that were genotyped, since the complete list more closely approximates the underlying mutational process than the genotyped list. We conclude that piRNA-generating loci are strongly biased towards regions of the genome with high rates of CNV formation and this observation was not explained solely by the fact that some piRNAs are repetitive.

It is important to note that although piRNAs are known to be strongly enriched in repeats in *Drosophila* [1] they are in fact *depleted* of repeats in mammals [10]. In particular, the piRNA data set that we used [10] does not distinguish between pachytene and pre-pachytene piRNA populations and so is not enriched for repeats, unlike some developmentally staged piRNA data sets [15]. Thus our comparison of rates of CNV formation in piRNA–generating loci versus repeat regions was conservative.

### The McDonald-Kreitman Test is Indicative of Negative Selection

Next we used a modified version of the McDonald-Kreitman test to study the evolution of human piRNA-generating loci at the CNV level. Traditionally, the McDonald-Kreitman test contrasts a putatively neutral class of sites (typically synonymous sites) to a putatively selected class of sites (typically nonsynonymous sites). For our study of CNV evolution, we instead used intergenic regions as the putatively neutral class of sites and piRNA-generating loci as the putatively selected class of sites.

The traditional McDonald-Kreitman test also contrasts divergence (typically fixed nucleotide substitutions between two species) to polymorphism (typically single nucleotide polymorphisms in one of the species). For our study, we used the CNVs from Conrad *et al.* [13] as the polymorphism data. We then computed the divergence data by sampling non-overlapping blocks of sequence from both piRNA-generating loci and intergenic regions and counting the number of times each block aligned to the reference human and chimpanzee genomes (Methods). Blocks that aligned a different number of times in human and chimpanzee were considered diverged. This procedure closely approximates the form of the polymorphism data that we used.

We computed a ratio of divergence to polymorphism of 1.97 for intergenic regions and 0.63 for piRNAs-generating loci (note that this ratio is not the same as the DN/DS ratio so a ratio >1 is not indicative of positive selection). This difference was highly statistically significant according to a Chi Square test (P-value <2.2 e−16). Also, the divergence of piRNA-generating loci (7.6%) was higher than that for intergenic regions (6%), consistent with a higher CNV mutation rate in piRNA-generating loci. Thus despite the strong enrichment of CNVs in piRNA-generating loci, there was a large excess of polymorphism in piRNA-generating loci compared to divergence, assuming that intergenic regions as a whole are evolving neutrally with respect to CNVs. We interpret this pattern as an indication of negative selection on piRNA-generating loci at the CNV level. Negative selection would cause an excess of mildly deleterious polymorphisms and a depletion of divergence, which is what we observed in the piRNA data. Note that if intergenic regions are under selective constraint with respect to CNVs, perhaps because of unannotated functional elements, that would only make our comparison more conservative.

One potential concern is that our procedure for estimating divergence might underestimate divergence in repetitive regions because repeats might be collapsed by the genome assembly algorithm into a single copy in both the human and chimp reference genome assemblies. In this case, divergence would not be detectable by sequence comparisons of the two genomes, but polymorphism would be detectable by tiling array methods [13]. However, since piRNAs are depleted of repeats in humans relative to intergenic regions as mentioned above, this would only make our inference of selective constraint on piRNA-generating loci stronger.

**Figure 1:**
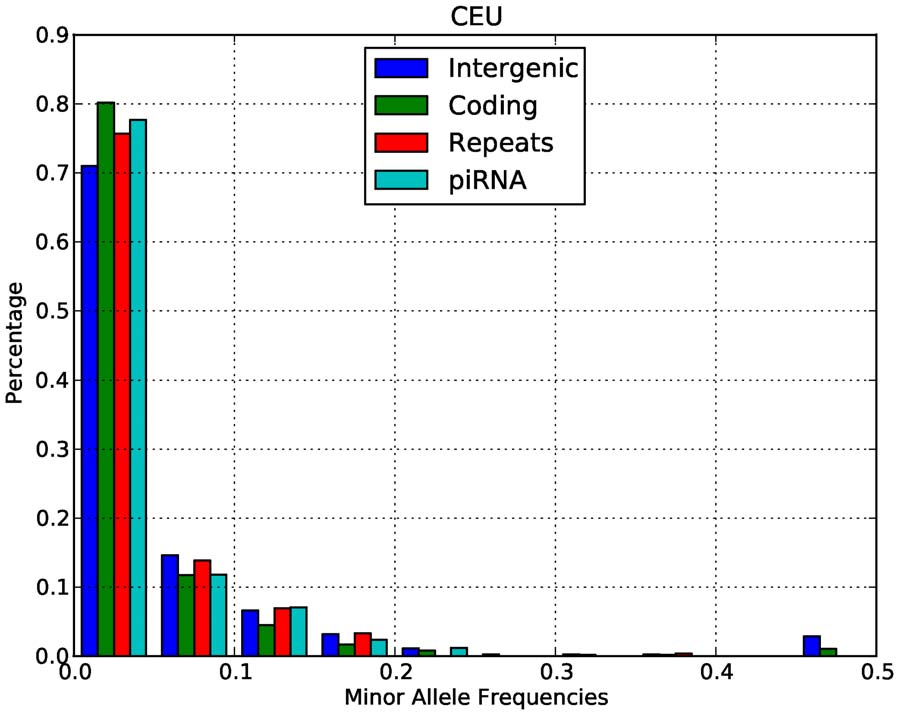
Minor allele frequency distributions in different classes of functional sites in the CEU population.

**Figure 2:**
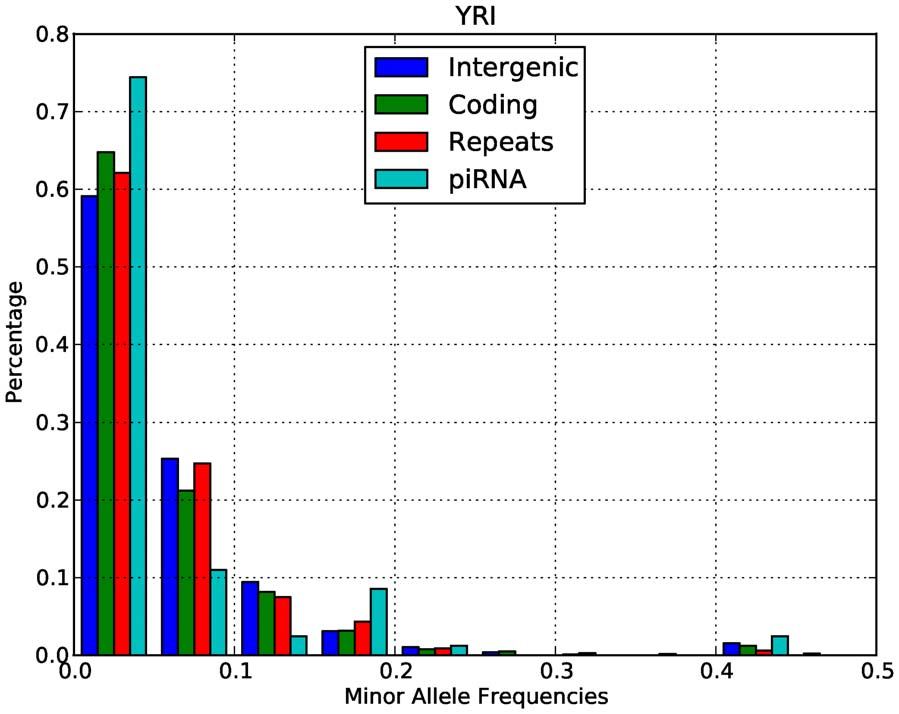
Minor allele frequency distributions in different classes of functional sites in the YRI population.

An alternative interpretation of our McDonald-Kreitman result is that there is balancing selection on CNVs, since this would also produce an enrichment of polymorphisms in piRNA-generating loci. Such a scenario would indeed be consistent with the role of piRNAs as an immune system defending against transposable elements. However, we do not observe an excess of alleles segregating at intermediate frequency in either Europeans (Figure 1) or Yorubans (Figure 2). This pattern is consistent with directional selection in both populations, not balancing selection. We also computed the genetic differentiation between Europeans and Yorubans in both the piRNA-generating loci and intergenic loci using the F_st_ statistic. Our F_st_ analysis showed that piRNA-generating loci in fact have lower F_st_ than intergenic regions (0.027 vs. 0.033), which is again consistent with global negative selection across populations, not balancing selection caused by local adaptation. We thus conclude that the primary selective force acting on piRNA-generating loci is negative selection.

So far we have considered all piRNA-generating loci in our analysis, without considering how they are clustered on the genome. To investigate the clustering property of piRNAs further, we clustered piRNA-generating loci using a similar method to a previous study (Methods) [10]. When we restricted our McDonald-Kreitman analysis to only the piRNA clusters, we found a similar pattern of selective constraint, with a ratio of divergence to polymorphism of 0.37. The slightly lower ratio of divergence to polymorphism for piRNA clusters vs. piRNA-generating loci on the whole is consistent with stronger selective constraint on piRNA clusters. This is reasonable since some individual piRNA-generating loci may be caused by false positive “piRNAs” that bound non-specifically to Piwi protein whereas we expect that piRNA clusters are more confidently defined.

We have considered only differences in copy number between human and chimp but not whether they are gains or losses. We considered aligning CNVs and genomic blocks to the macaque genome in order to root the gains and losses of sequence between human and chimpanzee. However, we found that this analysis was not possible because of the low coverage (5.1x) of the current macaque genome assembly [16]. Finally, note that it is statistically correct to use only polymorphism data from humans and not from chimps in the McDonald-Kreitman test. While some of the presumed “divergence” may in fact be polymorphic in chimps, no bias is introduced by considering only one chimp genome.

### The Minor Allele Frequency Spectrum is Indicative of Negative Selection

To confirm the results of the McDonald-Kreitman test, we studied the minor allele frequency distributions of CNV in Europeans and Yorubans. For this analysis we considered only biallelic CNVs in the data. Both the minor allele frequency distributions of Europeans (Figure 1) and Yorubans (Figure 2) were highly enriched for low allele frequency relative to intergenic regions (Wilcoxon test, P-value <2.2E−16). This pattern is again consistent with the action of negative selection on piRNA-generating loci relative to intergenic regions.

### The Derived Allele Frequency Spectrum Contains Significant Ancestral Allele Misspecification

In principle we could have misinterpreted the folded minor allele frequency distributions as indicative of negative selection instead of positive selection if the unfolded derived allele frequency distribution showed an excess of high frequency alleles, which would be consistent with the action of positive selection. To test this possibility, we attempted to root the CNVs in two different ways. In the first CNV rooting method, we classified each CNV as either a gain or a loss in humans based on an ancestral diploid copy number of two (i.e. a human allele >2 was considered a gain in humans and a human allele <2 was considered a loss in humans). We made this choice of ancestral allele because genotyping of seven chimpanzees revealed that the vast majority of human CNV loci are fixed for a diploid chimp allele of two (Supplementary Information in [13] and Donald Conrad, personal communication). Also, we used the program BLAT [17] to align each human CNV to the chimpanzee genome (Methods) and even at a liberal cutoff of 360 for the BLAT score, over 85% of human CNVs had one copy in the (haploid) chimpanzee genome, corresponding to a diploid copy number of two. In the second CNV rooting method, we tried to guard against the possibility that piRNA-generating loci are specifically biased towards regions of the genome where the convention of an ancestral allele of two is likely to fail. We thus used BLAT to determine the ancestral state of each CNV by aligning it to the chimpanzee genome and counting the number of copies present in the chimpanzee genome (Methods). Overall, both CNV rooting methods resulted in very similar derived allele frequency distributions (data not shown). However, since the BLAT ancestral allele inference procedure reduced the high frequency bin slightly, suggesting that it corrected some cases of ancestral allele misspecification, we used the BLAT ancestral calls for the remainder of the analysis.

**Figure 3:**
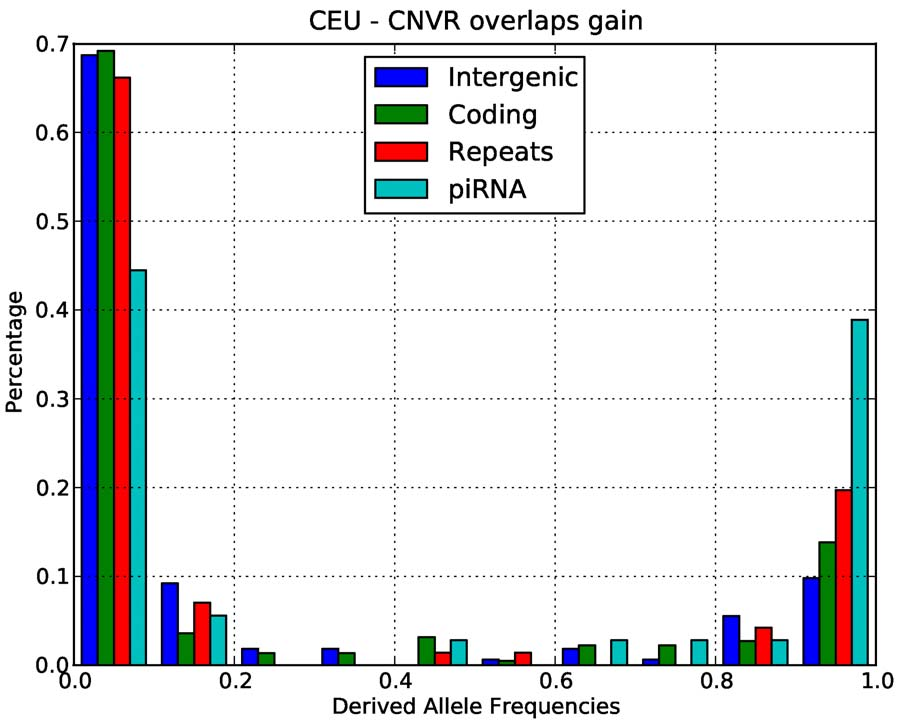
Derived allele frequency distributions for increased copy number in different classes of functional sites in the CEU population.

**Figure 4:**
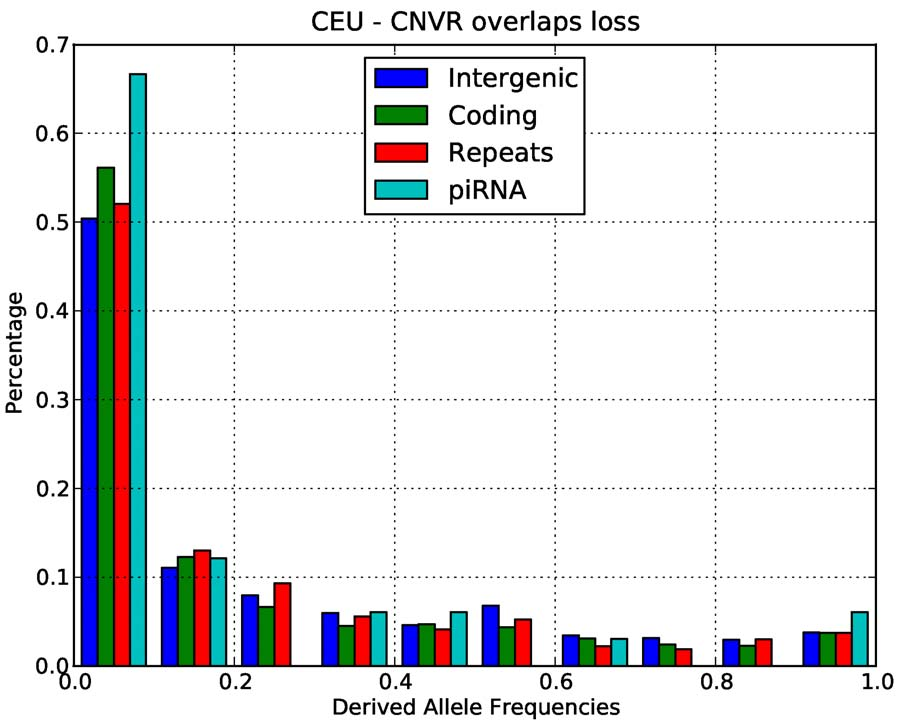
Derived allele frequency distributions for decreased copy number in different classes of functional sites in the CEU population.

Using the rooted CNV data, we produced the allele frequency spectrum for gains and losses in both Europeans and Yorubans. The data for gains in Europeans is shown in Figure 3 and the data for losses in Europeans is shown in Figure 4. The derived allele frequency distribution for losses is consistent with our interpretation of negative selection. The derived allele frequency distribution for gains had a very high excess of high frequency alleles and in principle this pattern might be consistent with the action of positive selection for gains. However, the derived allele frequency distributions departed significantly from the theoretical allele frequency distributions expected under positive selection. In particular, there was an excess of alleles only in the highest frequency bin and not at intermediate frequencies. According to the theoretical expectation, we would expect a gradual increase of alleles at intermediate frequency and only a small excess of alleles at the highest frequency bin. We thus believe that the pattern that we observed (Figure 3) is more likely to be due to widespread ancestral allele misspecification. This would be consistent with the high rate of CNV formation in piRNA-generating loci since we would expect a high rate of double mutations in these regions. In this case, the many of the CNVs in the highest frequency bin should be correctly assigned to the lowest frequency bin for losses. We conclude that the derived allele frequency spectrum did not contain convincing evidence for positive selection on piRNA CNVs but rather was consistent with our overall interpretation of negative selection.

## Discussion

In conclusion, we have studied the evolution of human Piwi-interacting RNA (piRNA) –generating loci at the copy number variation (CNV) level using a comprehensive set of CNVs genotyped in multiple human populations [13]. We showed that piRNA-generating loci have a very high CNV mutation rate, even compared with repeat regions of the genome. Our evolutionary analyses using the McDonald-Kreitman test and minor allele frequency distributions are indicative of negative selective pressure against copy number variation of piRNA-generating loci in humans. This pattern is consistent with the functional roles of piRNAs to repress transposable elements and negative selection on human piRNAs at the nucleotide substitution level [5]. While the derived allele frequency spectrum for CNV gains shows an apparent excess of high frequency alleles, we have argued that this pattern is more likely due to ancestral allele misspecification than positive selection.

Our results differ from the model proposed by Assis and Kondrashov [8] that there is positive selection for increased copy number of piRNA clusters in rodents. There are two possible explanations for this difference. The first explanation is that Assis and Kondrashov studied piRNAs in rodents whereas we studied piRNAs in primates and the history of transposable element insertions is known to be different in these two lineages. Notably, transposable elements in the rodent lineage have had a relatively high constant rate of insertion whereas in the primate lineage the insertion rates of transposable elements have been plummeting since a burst of Alu transpositions 40 million years ago [18]. The present rates of transposable element insertion are roughly four times higher in mouse than human [18]. Thus, one possibility is that there is positive selection for increased piRNA cluster number in rodents to defend against the relatively high transposition rate in that lineage but this pattern is not seen for the relatively inactive transposable elements in humans.

The second explanation for the difference in results is that Assis and Kondrashov only examined divergence data, not polymorphism data [8]. Thus it is possible that the pattern they observe is due to a high mutation rate and not the action of natural selection. This possibility would be consistent with our results in humans, since we observe a very high rate of CNV formation in piRNA-generating loci. However, this possibility can only be tested directly when appropriate CNV data from rodents becomes available.

## Materials and Methods

### Mapping piRNAs to the Genome

We mapped piRNAs to the human genome using two different read mapping programs. First, we used the program BWA [19], as previously described [5]. BWA allows multiply mapping reads but keeps a random location for each read. To remove the repetitive piRNAs, we also mapped the piRNA sequences using the program Bowtie [20], restricting only to uniquely mapping reads. We did not find a significant difference in the allele frequency distributions between the two mappings (data not shown) so we used the BWA mappings for the results presented in the manuscript.

To define piRNA clusters, we followed the thresholds of 20 kb and 90 kb in [10] to perform single-linkage clustering of piRNA-generating loci. The number of piRNA clusters with at least 10 piRNAs was very similar for either the 20 kb or 90 kb thresholds (152 or 143 respectively). We therefore used the 90 kb threshold for our analysis. Altogether 13,312 piRNAs are produced from piRNA clusters containing at least 10 piRNAs.

### Aligning Human CNVs and Genomic Blocks to the Chimpanzee Genome

To root the human CNVs, we downloaded the chimpanzee genome sequence from the UCSC Genome Browser [16]. We then used the program, BLAT [17], to align all human CNVs from the Conrad *et al.* data set [13] to the chimpanzee genome in a computationally efficient way. BLAT was designed to align mRNAs to the genome but the chimpanzee genome is similar enough to the human genome that we were able to use BLAT to align human genomic sequences to the chimpanzee genome. Although BLAT is optimized to run efficiently even with large genomes, we found that it performed slowly on some of the very long CNV sequences. To speed up the computations, we therefore truncated each CNV to 400 bases. This length is short enough to make the computations feasible but long enough to allow for confident matching of CNVs to the chimpanzee genome. We experimented with different BLAT scores, defined as the number of matches minus the number of mismatches, in the range of 360 to 390, in increments of 5. The results were similar (data not shown) so we chose a score of 375 for the results presented in this paper. A score of 375 is consistent with a combined substitution rate of ∼4% between human and chimpanzee (1% nucleotide substitution and 3% indel rate) [21].

To estimate the divergence between human and chimpanzee at the copy number level, we used the same score cutoff of 375 to count the number of BLAT hits in the chimpanzee genome for blocks of 400 bases centered on each piRNA. For computational tractability, we estimated the divergence for intergenic regions by sampling blocks of 400 bases and then extrapolating the rate of divergence to all intergenic regions. We removed all CNVs overlapping any known functional elements from the intergenic CNVs.

Since the chimpanzee genome is incomplete, we experimented with allowing a small difference between the chimpanzee state and the human alleles when determining the ancestral state. For example, when we allowed a difference of 1, if the human alleles were 0 and 1 and the chimp allele was 2 we assigned 1 as the ancestral allele. We found that allowing any difference greater than 0 resulted in much more noisy derived allele frequency distributions, so we confined our analysis to just assigning exact chimpanzee state as the ancestral allele. Any CNV for which all human alleles failed to match the chimpanzee allele was discarded.

## Supporting Information

Figure S1 **Derived allele frequency distributions for decreased copy number in different classes of functional sites in the CHBJPT population.**
(EPS)

## References

[1] Siomi M, Sato K, Pezic D, Aravin A (2011) PIWI-interacting small RNAs: the vanguard of genome defence. Nat Rev Mol Cell Biol 12: 246–258. 21427766 10.1038/nrm3089

[2] Brennecke J, Aravin A, Stark A, Dus M, Kellis M, et al (2007) Discrete small RNA-generating loci as master regulators of transposon activity in Drosophila. Cell 128: 1089–1103. 17346786 10.1016/j.cell.2007.01.043

[3] Kumar M, Chen K (2012) Evolution of Animal Piwi-interacting RNAs and Prokaryotic CRISPRs. Brief Funct Genomics. 10.1093/bfgp/els016 PMC3398257 22539610

[4] Grimson A, Srivastava M, Fahey B, Woodcroft B, Chiang H, et al (2008) Early origins and evolution of microRNAs and Piwi-interacting RNAs in animals. Nature 455: 1193–1197. 18830242 10.1038/nature07415 PMC3837422

[5] Lukic S, Chen K (2011) Human piRNAs are under selection in Africans and repress transposable elements. Mol Biol Evol 28: 3061–3067. 21613236 10.1093/molbev/msr141 PMC3199439

[6] Ewing A, Kazazian HJ (2011) Whole-genome resequencing allows detection of many rare LINE-1 insertion alleles in humans. Genome Res 21: 985–990. 20980553 10.1101/gr.114777.110 PMC3106331

[7] Lu J, Clark A (2010) Population dynamics of PIWI-interacting RNAs (piRNAs) and their targets in Drosophila. Genome Res 20: 212–227. 19948818 10.1101/gr.095406.109 PMC2813477

[8] Assis R, Kondrashov A (2009) Rapid repetitive element-mediated expansion of piRNA clusters in mammalian evolution. Proc Natl Acad Sci USA 106: 7079–7082. 19357307 10.1073/pnas.0900523106 PMC2667148

[9] Ruby J, Jan C, Player C, Axtell M, Lee W, et al (2006) Large-scale sequencing reveals 21U-RNAs and additional microRNAs and endogenous siRNAs in C. elegans. Cell 127: 1193–1207. 17174894 10.1016/j.cell.2006.10.040

[10] Girard A, Sachidanandam R, Hannon G, Carmell M (2006) A germline-specific class of small RNAs binds mammalian Piwi proteins. Nature 442: 199–202. 16751776 10.1038/nature04917

[11] Kolaczkowski B, Hupalo D, Kern A (2011) Recurrent adaptation in RNA interference genes across the Drosophila phylogeny. Mol Biol Evol 28: 1033–1042. 20971974 10.1093/molbev/msq284

[12] Castillo D, Mell J, Box K, Blumenstiel J (2011) Molecular evolution under increasing transposable element burden in Drosophila: a speed limit on the evolutionary arms race. BMC Evol Biol 11: 258. 21917173 10.1186/1471-2148-11-258 PMC3185285

[13] Conrad D, Pinto D, Redon R, Feuk L, Gokcumen O, et al (2010) Origins and functional impact of copy number variation in the human genome. Nature 464: 704–712. 19812545 10.1038/nature08516 PMC3330748

[14] Smit A, Hubley R, Green P (1996–2010) RepeatMasker Open-3.0. http://www.repeatmasker.org. Accessed through the UCSC Genome Browser (version 3.2.7 Jan 2009).

[15] Aravin A, Sachidanandam R, Girard A, Fejes-Toth K, Hannon G (2007) Developmentally Regulated piRNA Clusters Implicate MILI in Transposon Control. Science 316: 744–747. 17446352 10.1126/science.1142612

[16] Dreszer T, Karolchik D, Zweig A, Hinrichs A, Raney B, et al (2012) The UCSC Genome Browser database: extensions and updates 2011. Nucleic Acids Red 40: D918–923. 10.1093/nar/gkr1055 PMC3245018 22086951

[17] Kent W (2002) BLAT–the BLAST-like alignment tool. Genome Res 12: 656–664. 11932250 10.1101/gr.229202 PMC187518

[18] Mouse Genome Sequencing Consortium, Waterston R, Lindblad-Toh K, Birney E, Rogers J, et al (2002) Initial sequencing and comparative analysis of the mouse genome. Nature 420: 520–562. 12466850 10.1038/nature01262

[19] Li H, Durbin R (2009) Fast and accurate short read alignment with Burrows-Wheeler transform. Bioinformatics 25: 1754–1760. 19451168 10.1093/bioinformatics/btp324 PMC2705234

[20] Langmead B, Trapnell C, Pop M, Salzberg S (2009) Ultrafast and memory-efficient alignment of short DNA sequences to the human genome. Genome Biol 10: R25. 19261174 10.1186/gb-2009-10-3-r25 PMC2690996

[21] Chimpanzee Sequencing, Analysis Consortium (2005) Initial sequence of the chimpanzee genome and comparison with the human genome. Nature 437: 69–87. 16136131 10.1038/nature04072

