# Supplementary figures and images for: Selective Constraint on Copy Number Variation in Human Piwi-Interacting RNA Loci
Source: PLoS One. 2012 Oct 4;7(10):e46611. doi: 10.1371/journal.pone.0046611 (PMC3464240; doi:10.1371/journal.pone.0046611)

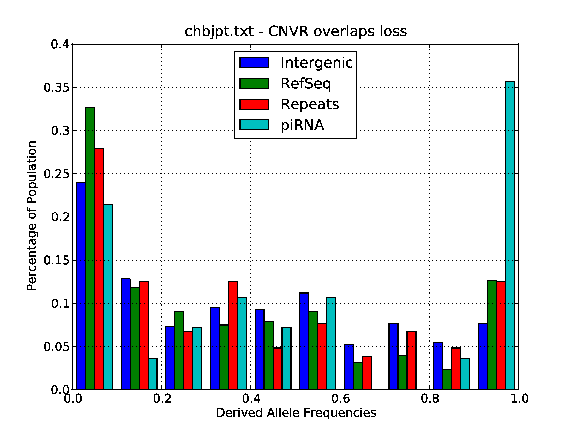

Supplement: Figure S1 — Derived allele frequency distributions for decreased copy number in different classes of functional sites in the CHBJPT population. (EPS) [file pone.0046611.s001.tif]
